# Performance and confusion effects for gist perception of scenes: An investigation of expertise, viewpoint and image categories

**DOI:** 10.1177/03010066251345677

**Published:** 2025-06-24

**Authors:** Emil Skog, Andrew J. Schofield, Timothy S. Meese

**Affiliations:** School of Psychology, College of Health and Life Sciences, 1722 Aston University, UK; Aston Laboratory for Immersive Virtual Environments, College of Health and Life Sciences, 1722 Aston University, UK; Department of Health, Learning and Technology, 5185 Luleå University of Technology, Sweden; School of Psychology, College of Health and Life Sciences, 1722 Aston University, UK; Aston Laboratory for Immersive Virtual Environments, College of Health and Life Sciences, 1722 Aston University, UK; Aston Laboratory for Immersive Virtual Environments, College of Health and Life Sciences, 1722 Aston University, UK

**Keywords:** visual expertise, categorisation, features/parts, object recognition, scene perception, viewpoint, gist perception

## Abstract

Human object recognition often exhibits viewpoint invariance. However, unfamiliar aerial viewpoints pose challenges because diagnostic features are often obscured. Here, we investigated the gist perception of scenes when viewed from above and at the ground level, comparing novices against remote sensing surveyors with expertise in aerial photogrammetry. In a randomly interleaved single-interval, 14-choice design, briefly presented target images were followed by a backward white-noise mask. The targets and choices were selected from seven natural and seven man-made categories. Performance across expertise and viewpoint was between 46.0% and 82.6% correct and confusions were sparsely distributed across the 728 (2 × 2 × 14 × 13) possibilities. Both groups performed better with ground views than with aerial views and different confusions were made across viewpoints, but experts outperformed novices only for aerial views, displaying no transfer of expertise to ground views. Where novices underperformed by comparison, this tended to involve mistaking natural for man-made scenes in aerial views. There was also an overall effect for categorisation to be better for the man-made categories than the natural categories. These, and a few other notable exceptions aside, the main result was that detailed sub-category patterns of successes and confusions were very similar across participant groups: the experimental effects related more to viewpoint than expertise. This contrasts with our recent finding for perception of 3D relief, where comparable groups of experts and novices used very different strategies. It seems that expertise in gist perception (for aerial images at least) is largely a matter of degree rather than kind.

## Introduction

The ability to recognise the same object from multiple viewpoints is a general property of human object recognition (Marr, 1982). Nevertheless, unfamiliar viewpoints, where key object features and/or shapes are not explicit in the retinal image, introduce difficulties for human observers. For many objects, this happens when they are viewed from above or end-on (e.g., Newell & Findlay, 1997). For example, ground views of most houses will contain image information about walls, doors, windows and the roof, including its slope, but an aerial photograph might depict only the roof and impoverished cues to its 3D relief. In general, aerial viewpoints radically change the appearances of landscape features compared to ground-views, making most aerial scenes more difficult to process and classify (Lloyd et al., 2002; Loschky et al., 2015; Oliva & Torralba, 2001; Pannasch et al., 2014). This was illustrated by Pannasch et al. (2014), for example, who compared the amplitudes, durations and individual differences of eye-movements across viewpoints and concluded that the information processing effort was greater for aerial scenes than for ground-view scenes.

A situation where objects and scenes are frequently seen from above is in aerial photogrammetry where remote sensing surveyors use photographs taken from aircraft as the basis for map-making. Surveyors undergo specific training for this and gain expertise over time which enhances their visual processing skills (Borders et al., 2020; Harel, 2016; Lansdale, Underwood & Davies, 2010; Lloyd et al., 2002; Seitz, 2020; Skog et al., 2024; Šikl et al., 2019). For example, in a visual search task, Lansdale et al. (2010) found that novices were consistently drawn to salient features in aerial images, whereas expert remote sensing surveyors employed by Ordnance Survey (OS, the Government national mapping agency for Great Britain) were able to discount these when irrelevant. Experts also made different patterns of eye-movements compared to novices in expert-relevant visual tasks (Bertram et al., 2013; Krupinski et al., 2006; O’Neill et al., 2011; see Fox & Faulkner-Jones, 2017, for a review).

As part of a larger project working with the OS on visual perception and cognition of aerial scenes, we wanted to better understand the visual techniques and processes used by expert remote sensing surveyors in aerial recognition tasks. To do this, we identified three aspects of the surveyors’ work for which relevant paradigms in visual perception and cognition have been developed: depth perception, scene gist recognition and mental rotation. We review our work on depth perception (Skog et al., 2024) below and in the General Discussion, and present our investigation of mental rotation in a companion paper (Skog et al., under review); here, we consider the gist recognition of a scene.

### Gist Perception

In general, most participants are remarkably fast and accurate in identifying the gist of a briefly presented scene (Castelhano & Henderson, 2008; Davenport & Potter, 2004; Fei-Fei et al., 2007; Greene & Oliva, 2009; Malcolm et al., 2016; Oliva & Torralba, 2006; Rayner et al., 2009; Rousselet et al., 2005; Schyns & Oliva, 1994), with superordinate category discriminations between natural and man-made scenes taking place for viewing times as short as 16 ms (Furtak et al., 2022; Joubert et al., 2007; Loschky & Larson, 2010). Furthermore, the superordinate distinction of scenes as either natural or man-made seems to precede finer level distinctions (e.g., street or city centre: Loschky & Larson, 2010).

While several studies of gist perception have concentrated on basic categories such as ‘mountain’, ‘forest’ and ‘street’, some have considered the relative salience of superordinate categories (such as indoor/outdoor; natural/man-made). Fei-Fei et al. (2007) found a bias towards describing scenes as outdoor in a free recall paradigm, and Loschky and Larson (2008) found a bias towards natural versus man-made for phase randomised stimuli. In contrast, using a go/no-go task, Joubert et al. (2007) showed a modest processing advantage for man-made versus natural scenes, finding that the minimal reaction time (the shortest RT associated with a significant difference between correct and incorrect ‘go’ responses) was significantly shorter for man-made scenes although median RTs did not differ between these superordinate categories.

Although the perception of gist by novice observers sets a high bar (Drew et al., 2013), perceptual expertise can raise it even higher. For example, radiologists outperform novices considerably in detecting small abnormal targets in briefly presented X-ray images (∼250 ms) (Bertram et al., 2013; Drew et al., 2013). Furthermore, and consistent with anecdotal reports that radiologists know something is wrong before they know where it is, Evans et al. (2013) found that radiologists were unable to pinpoint the locations of the abnormalities they detected. This suggests an underlying process for gist that is sensitive but unselective for small details/objects in the scene (i.e., it has poor spatial resolution), relying instead on global image measures (Chin et al., 2018; Drew et al., 2013; Kundel & Nodine, 1975), consistent with a course-to-fine timeline for gist processing (Schyns & Oliva, 1994). This raises the intriguing possibility that visual experts might have or develop a special facility for global image content. On the other hand, Evans et al. (2013) were criticised on methodological grounds in a failure to replicate (Carrigan et al., 2018) and psychophysical investigation of university attendees found no evidence for a decoupling between detection and localisation (Carrigan et al., 2019).

### Aims and Antecedents

In view of all the above, we hypothesised that the expertise of remote sensing surveyors might benefit their perception of gist for aerially viewed scenes. In recent work using the classification image (CI) technique (Skog et al., 2024), we found that these experts were better than novices at discriminating aerial views of hedge-like stimuli from ditch-like stimuli and made better use of binocular disparity cues when doing this. Furthermore, it was not that the experts had (or had developed) better stereoacuity but were better at constructing 3D relief from the disparity cues (see also, Carrillo et al., 2020). On the other hand, and surprisingly, novices paid more tacit attention to luminance cues than did the experts, pointing to a difference in perceptual strategy, not just a difference in proficiency across groups. Skog et al. (2024) ran their study using a short stimulus duration (750 ms) and a simultaneous noise mask. Although longer than typical stimulus processing times in gist studies, it is much shorter than the unlimited image inspection time available to photogrammeters in the workplace. This makes it likely that our previous work was tapping a fundamental aspect of expert visual processing not just a difference in ‘offline’ cognitive strategy. Taking all these points together, we wondered whether interesting differences might emerge across novices and experts for the rapid (100 ms) scene gist recognition task investigated here, particularly in terms of category confusions.

We note three previous studies directly relevant to this one. Lloyd et al. (2002) compared aerial scene categorisations by university geography- and non-geography students and found the geographers were more accurate and faster than non-geographers. Extending this work to a memory task, Šikl et al. (2019) found professional remote sensing surveyors were even more accurate than geography students. However, neither of those studies limited visual processing time, nor did they compare ground with aerial scenes. These matters were tackled by Loschky et al. (2015) who found that rapid visual categorisation was less accurate (by about 15–20%) for aerial-views compared to ground-views. From their image analysis and a further experiment in which they presented the images at various orientations, they also concluded that the performance detriment for aerial-views derived from a dependence on rotation-independent features which were less valuable than the gravity-specific configuration cues available in ground-based images. However, Loschky et al. did not compare novices and experts. We extended the studies above by using an approach similar to Loschky et al. but comparing novices with professional experts. In further refinements of Loschky et al.'s work, we also: (i) used more image categories (fourteen compared to ten, increasing the number of potential cross-category confusion types from 90 to 182), (ii) considered performance at both the basic and superordinate category levels and (iii) performed a preliminary assessment of the rank order of the image categories along a natural-man-made dimension. This ranking task was designed to enhance the visualisation of results in confusion matrices (CMs) by bringing meaning (dimensional proximity) to the spatial proximity of cells in the matrices.

Finally, there is a general problem in trying to investigate the influence of visual expertise on gist perception, since typical adult participants have general expertise in the categorisation of most real-world scenes. The study here provides a solution to this problem by identifying a set of scenes (aerial views) for which only a small subset of participants (remote sensing surveyors) has the relevant expertise (Loschky, personal communication).

## Method

### Stimuli

Ground-view images were sourced from the public domain on Flickr (www.flickr.com) while aerial images were sourced from OS. The distance from the camera to the subject of the photograph was not controlled for the ground view images, but typically it was closer than for the aerial images. To bring uniform diversity to the scenes, 14 sub-categories were chosen from an even mix of ‘natural’ and ‘man-made’ superordinate categories. For each of the sub-categories there were ten ground-view and ten aerial images giving a total of 280 images. The expert participants were recruited from OS, but scene categories were not selected or distinguished by common OS task specifications. For example, the experts would be accustomed to categorising both ‘crop field’ and ‘cattle field’ as ‘agricultural’.

All images were cropped to a square, converted to greyscale, down-sampled to 384 × 384-pixels using bicubic interpolation and stored in Portable Network Graphics format using MATLAB (The MathWorks Inc). During the experiment, images were scaled in PsychoPy (Peirce et al., 2019) using linear interpolation to a square set to 30% of the participant's monitor height in pixels.

**Figure 1.**
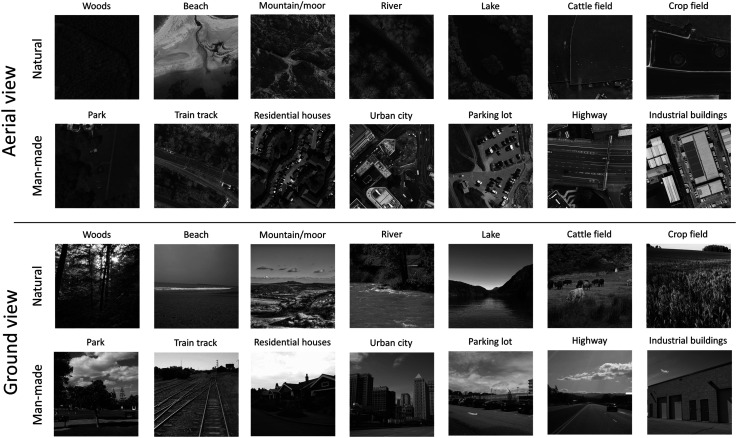
Example Stimuli from all 14 scene categories for ground and aerial viewpoints. (Aerial views: *© Crown copyright and database rights 2025 OS*, used with permission; ground views: public domain www.flickr.com). The full stimulus set contained 10 exemplars from each category (a total of 280 different images).

As part of the stimulus selection process, two naïve participants and authors ES and AJS judged whether each stimulus was unambiguously consistent with its scene category label. These judges did not participate in the experiment. To satisfy these judges, two ground-view images and nine aerial images were replaced. (An example of an excluded image was where a ‘parking lot’ was in close proximity to ‘industrial buildings’.) Representative examples of the final set of stimuli are shown in Figure 1.

In a preliminary survey conducted online via Prolific (www.prolific.co), twenty experimentally naïve participants who were native English-speakers and based in the UK, rated the image categories from most-to-least natural. The survey provided only the text of the 14 sub-category names; no images were provided for reference. Respondents ranked the scenes in the following order from most-to-least natural: woods, beach, mountain/moor, river, lake, cattle field, crop field, park, train track, residential houses, urban city, parking lot, highway and industrial buildings, consistent with our initial superordinate distinction between natural and man-made (see Figure 1).

### Materials

The experiment was created using PsychoPy and JavaScript to run on the online experiment delivery platform Pavlovia (Pavlovia.org). Participants used their own desktop computers to access and run the experiment by clicking a hyperlink that opened the experiment in their web-browser. The computer, monitor, mouse and keyboard, viewing distance and testing environment were not controlled. PsychoPy handled stimulus timings (PsychoJS, version 2021.2.0). Monitor framerates were recorded.

### Participants

The experiment was advertised via email to the expert participants and via Prolific to the novice participants. Fourteen expert participants were recruited from OS [7 female; mean age 40 years (*SD* = 12); mean experience of remote sensing surveying = 7 years (*SD* = 6, range: 1–25 years)]. Fifteen novice participants were recruited from Prolific, but one was excluded for failing an attention check (see *Procedure* for details) [7 female; mean age: 37 years (*SD* = 10)]. The novices averaged 737 (*SD* = 538) total approved participations in previous studies and surveys on Prolific. All participants were fluent or native speakers of English and were based in the UK or Ireland. All participants were compensated financially at a rate of £10 an hour. The number of novice participants recruited was determined by the number of available expert participants prepared to volunteer their time.

### Procedure

The project was reviewed by Aston University's College of Health and Life Sciences Ethical Review committee (approval number 1843) and all participants gave informed consent via a button press. All participants carried out the experiment during daytime hours. By way of a button press, all experts indicated they had significant experience with aerial images and all novices indicated they had not.

The experiment began with an explanation of the task: to identify scene categories in briefly presented scenes seen from both ground and aerial viewpoints. Example images from the scene categories, ‘crop field’ and ‘residential houses’ were shown with unlimited presentation time and from both viewpoints to familiarise participants with ground- and aerial-views. The initial text screen also listed the fourteen response options and described how to make responses by clicking buttons on the screen with the computer mouse. The buttons were arranged in two rows with an alternating mix of natural and man-made scenes in each row. The order was as follows: Top row: Woods, Train track, Crop field, Residential houses, Lake, Highway, River; Bottom row: Industrial buildings, Mountain/moor^1^, Parking lot Cattle field, Park, Beach and Urban city. The instructions promoted careful attention to the task and emphasised that the images would be presented very briefly. Participants then continued to the practise trials.

Each trial sequence was as follows: (i) a blank screen (1,000 ms), (ii) a central small black fixation cross to prompt attention (1,000 ms)—attention is important for perception of gist (Mack & Clarke, 2012) –, (iii) removal of the fixation cross and presentation of a central target image (100 ms—six image frames on a 60 Hz monitor^2^), (iv) a central white noise texture (32 × 32 elements) serving as a backwards mask (100 ms), (v) a blank screen (500 ms) and (vi) a time-unlimited presentation of the fourteen response option buttons placed just below the centre of the screen and extinguished by the participant's response.^3^ Each new trial was initiated automatically.

The experiment began with 20 practice trials, which included ten ground-view and ten aerial images from different sub-categories. These images were the same for all participants but were not used in the main experiment. The 280 test stimuli were presented in a different interleaved random order for each participant. After every 70th trial (i.e., four times in total), an ‘attention check’ text string was presented in the middle of the screen for one second. This said one of: ‘Red’, ‘Green’ or ‘Blue’. Three buttons then appeared with corresponding text labels prompting a matching response. A pause screen followed each attention check, affording a self-timed break and an indication of progress through the experiment. The total time for completing the experiment was around 25 min.

## Results and Discussion

### Accuracy of Scene Categorisation

**Figure 2.**
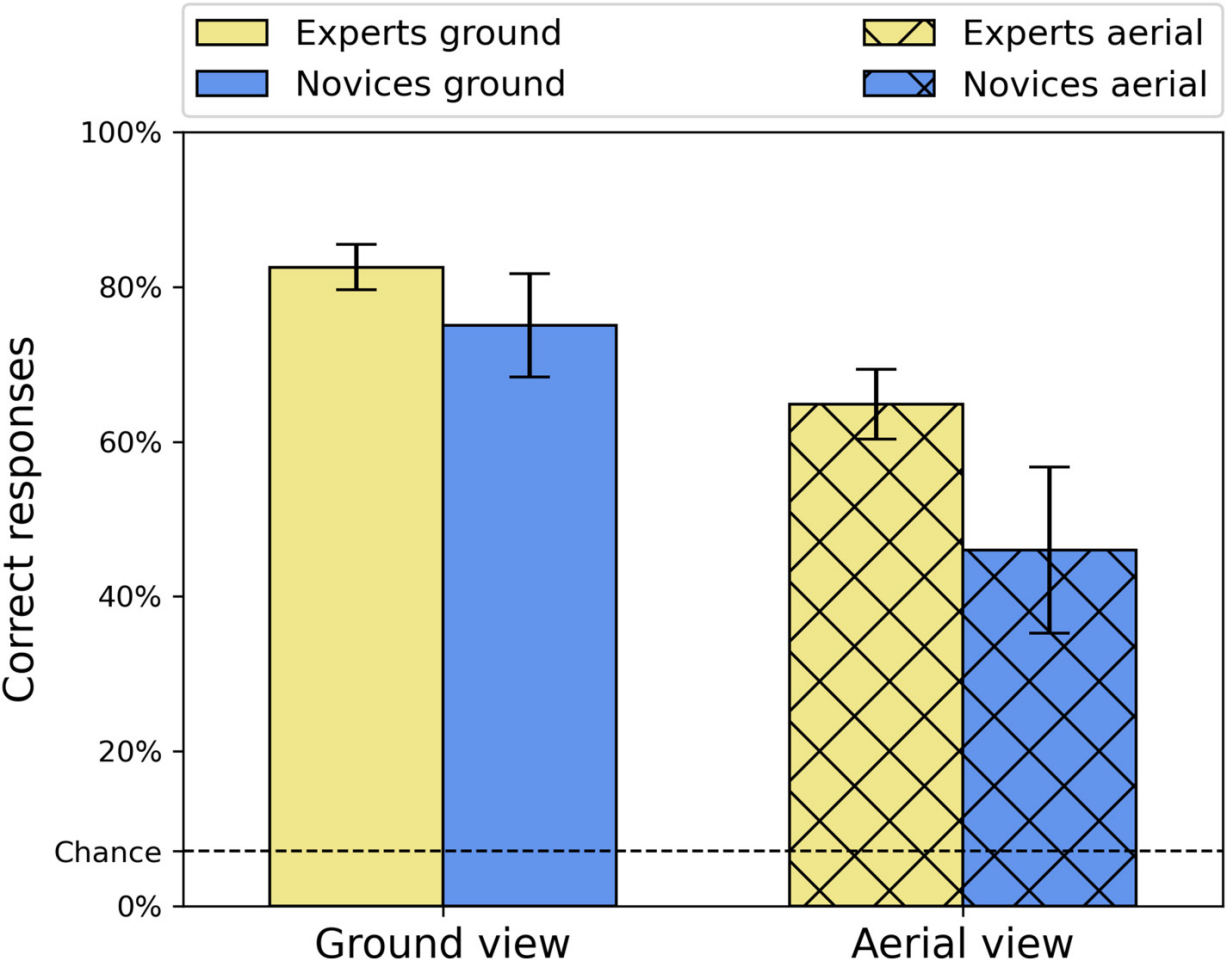
Average accuracies (percent correct) for the two groups and two viewpoint conditions. Error bars are 95% confidence intervals.

Accuracy (% correct) is shown in Figure 2 for both participant groups (novice, expert) and both viewpoint conditions (ground, aerial) and shows no signs of ceiling or floor effects. A Shapiro–Wilk test confirmed normality of the data and a 2 × 2 repeated-measures ANOVA found significant main effects for viewpoint [*F*(1, 26) = 202.0, *p* < .001, *η*^2^_G_ = 0.46 and group (*F*(1, 26) = 7.98, *p* = .009, *η*^2^_G_ = 0.21] and a significant interaction between the two [*F*(1, 26) = 11.9, *p* = .002, *η*^2^_G_ = 0.048]. Tukey corrected Post-hoc *t*-tests showed that experts made significantly more correct responses than novices for the aerial images [*t*(26) = 3.18, *p* = .019], but not for ground-view images [*t*(26) = 2.02, *p* = .206]. Furthermore, and as expected (e.g., Loschky et al., 2015), the within-group accuracy was significantly higher for the ground-view images than the aerial images for both the experts [*t*(26) = 7.61, *p* < .001] and the novices [*t*(26) = 12.49, *p* < .001].

### Analysis of Correct Responses Per Category and the Problem of Bias

**Figure 3.**
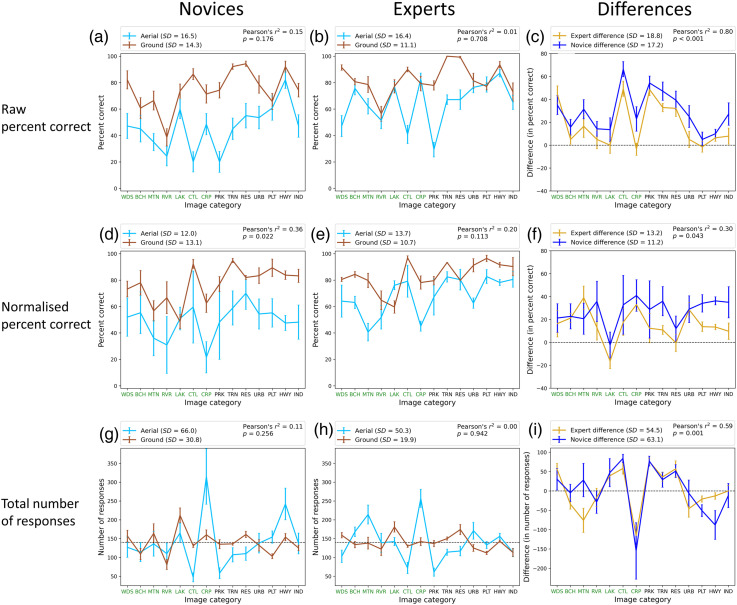
Analysis of correct responses per category for the two participant groups. (a and b) Percent correct for ground and aerial views for novices and experts, respectively. (c) The differences in percent correct (ground—aerial) between the two viewpoints for experts and novices. (d, e and f) The same as (a, b and c) but for normalised responses (see main text). (g and h) The total number of responses for each category for novices and experts, respectively. (i) The differences in the total number of responses between the two viewpoints for experts and novices. The natural- and man-made category divisions are denoted by labels in green and black, respectively (see online version of manuscript for full colour). Error bars are ±1SE across participants. The correlations in the legends (*r*^2^) are for the pair of data sets (different colours) in each panel. Axis label codes are: WDS: woods; BCH: beach; MTN: mountain/moor; RVR: river; LAK: lake; CTL: cattle field; CRP: crop field; PRK: park; TRN: train track; RES: residential buildings; URB: urban city; PLT: parking lot; HWY: highway; IND: industrial buildings.

The analysis above tells us about the main features of the results, but we wondered whether there were any deviations from these at the category level. Figure 3 shows the total number of responses, percent correct and normalised percent-correct (see below) for each viewpoint, group and category as well as differences between the viewpoints for each group and category. The categories are presented in rank order of naturalness determined by the preliminary survey with labels set in green and black for natural and man-made scenes, respectively. A comparison for the novices (Figure 3a) shows no image-category where the hit rate was higher when viewing was from above compared to the ground. The same comparison for experts (Figure 3b) shows they made slightly more correct responses when identifying crop fields (CRP) and parking lots (PLT) from above, but these differences were not significant. Similarly, a comparison across groups (not shown) found that experts were more often correct than novices for every category from both viewpoints, except industrial buildings (IND) at the ground level but again, this marginal difference was not significant.

There are marked variations in percent correct across categories in Figure 3a and b, but these were not correlated across viewpoints for either novices or experts [Pearson's correlations. Novices: *r*^2^(12) = .15, *p* = .176; experts: *r*^2^(12) = .01, *p* = .708]. On the other hand, percent correct was correlated across group for both viewpoints [ground: *r*^2^(12) = .85, *p* < .001; aerial: *r*^2^(12) = .7, *p* < .001; not shown] and the differences across viewpoint also showed a similar pattern against category across groups [Figure 3c; *r*^2^(12) = .8, *p* < .001]. This implies that although experts performed better than novices for aerial views, the difficulties experienced when switching to this viewpoint had specific links to the image categories and these were similar across groups. However, because this analysis is based on percent correct scores across categories it is possible that it is contaminated by bias.

Setting out to address the bias issue above, Figure 3g and h show the total number of responses per category for the different views across group and confirms that responses were not evenly distributed across category for either group, particularly for the aerial images where the bias towards crop fields (CRP) was markedly strong. The finding that bias changed substantially across viewpoints (e.g., CRP in Figure 3g and h, and HWY in Figure 3g) suggests a perceptual factor to the bias, a loss of diagnostic information for the aerial discriminations being a plausible possibility (Loschky et al., 2015). This is supported by the general lack of correlation across viewpoints for both groups [Figure 3g and h; novices: *r*^2^(12) = .11, *p* = .256; experts: *r*^2^(12) = .0, *p* = .942]. On the other hand, the correlations across experts and novices reveal a common bias factor [ground: *r*^2^(12) = .65, *p* = .001; aerial: *r*^2^(12) = .6, *p* = .001; not shown]. Furthermore, these biases changed with viewpoint in a similar way for each group [Figure 3i; *r*^2^(12) = .59, *p* = .001], and not just for crop fields (CRP), though mountain/moor (MTN) and highways (HWY) were obvious outliers. We return to these outlying categories further below.

The last correlation above points again to an image/perception-based factor for the biases though our data cannot rule out an interaction of this with response bias. We note that randomising the location of the response labels would have ruled out a contribution from a positional form of response bias (cf. Loschky et al., 2015). However, we did not design our experiment this way because (i) we were concerned that the resulting visual search might have been disruptive for our participants with our fourteen categories and we did not want task-irrelevant cognitive load to interfere with our results and (ii) this control would not rule out other forms of response bias; a simple aversion for cattle fields (CTL) for example, which we cannot control (or know about).

Putting aside whether response bias contributed to the category biases, the responses across categories (Figure 3g‒i) provide good evidence for an image-based perceptual effect of viewpoint on the results (for the reasons discussed above), but one that is not substantially tied to the expertise of the participant group.

### Mitigating for Bias in % Correct by Normalisation

To get closer to an estimate of genuine observer sensitivity to the targets, we followed Hansen and Loschky's (2013) approach for handling bias in a single-interval many-choice task (see also Loschky et al., 2015). We treated each 2 × 2 arrangement of viewpoint and participant group independently (the four data sets in Figure 3a and b), dividing each percent correct score by the sum of the percentage responses for that category (within the data set) across each stimulus category, and then multiplying by 100 to derive a normalised score for percent correct. These are shown in Figure 3d and e and confirm the superiority of experts over novices and the benefit of the ground views over aerial views. They also show that when bias is mitigated, sensitivity remained uneven across categories; a weak correlation across viewpoints became evident for the novices [novices: *r*^2^(12) = .36, *p* = .022; experts: *r*^2^(12) = .2, *p* = .113] but was less than that found across group for both viewpoints [ground: *r*^2^(12) = .75, *p* < .001; aerial: *r*^2^(12) = .61, *p* < .001; not shown]. With this metric, the differences across viewpoints are quite similar across categories (the curves in Figure 3f are quite flat; *SD* = 11.2% for novices, *SD* = 13.2% for experts), the obvious outliers being lakes (LAK), which we return to below, and possibly residential buildings (RES). This low-level variance across categories within group contributed to a low (but significant) correlation of the differences across groups [*r*^2^(12) = .3, *p* = .043; Figure 3f]. Nonetheless, the high correlations for % correct differences pre-normalisation (Figure 3c) and for number of responses (Figure 3i), indicate that the category dependent changes of response across viewpoint is largely independent of expertise.

### Superordinate Category Comparisons

**Figure 4.**
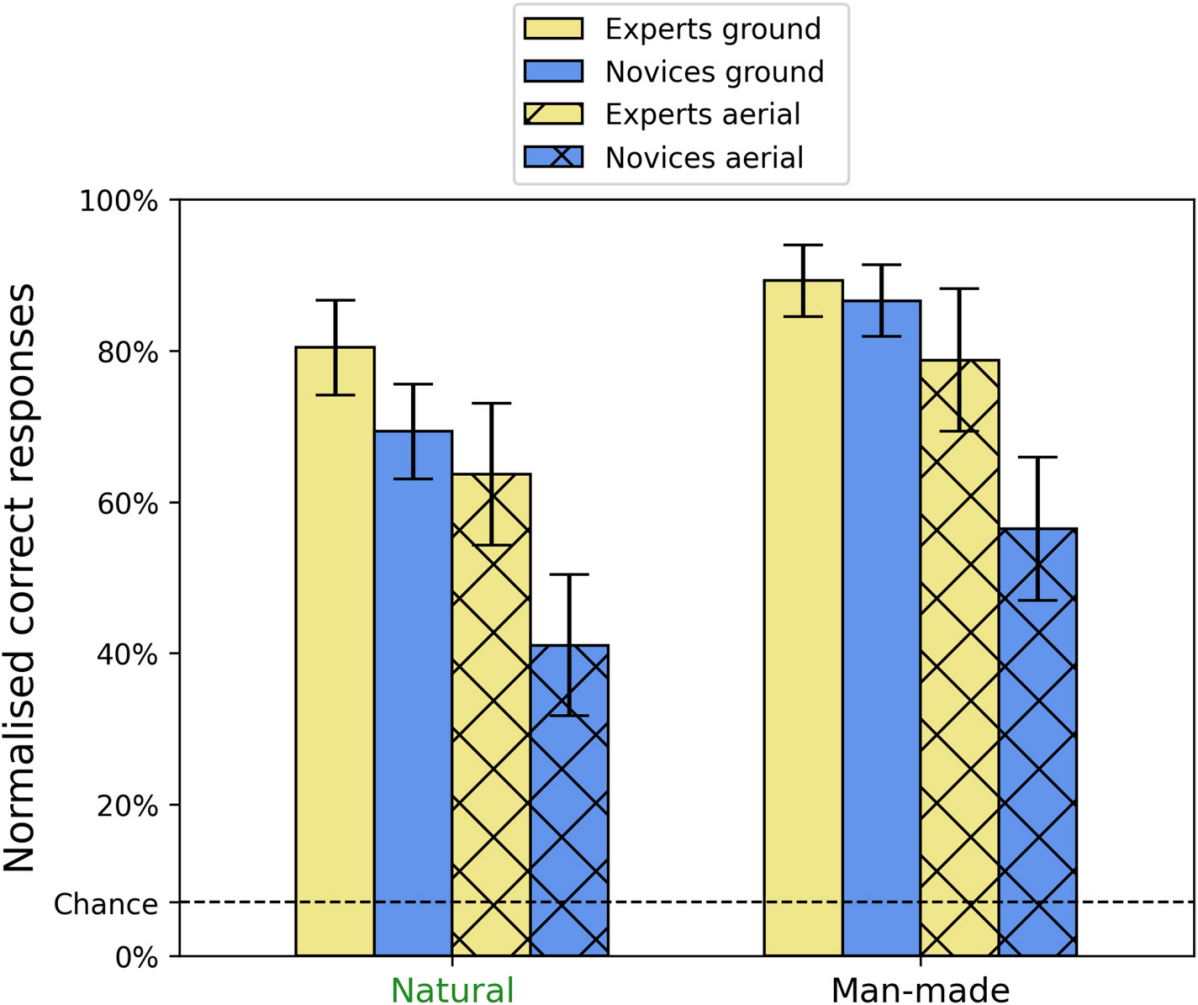
Mean normalised percent correct scores for the three-way interaction between group, viewpoint and superordinate category. Error bars show 95% confidence intervals.

Stepping back, we next considered normalised sensitivity (see above) for the superordinate categories (natural vs. man-made) for each of the 2 × 2 arrangements of viewpoint and group (see Figure 4). It was critical to use the normalised results here because potential differences might otherwise owe to pure categorical bias (see Figure 3g and h). One element of this comparison (experts viewing man-made scenes from the ground) was not normally distributed, having negative skew, so we raised all the data to the power 2, thus improving this distribution. A 2 × 2 × 2 mixed design ANOVA on the transformed data showed significant main effects for viewpoint [*F*(1, 26) = 259.66, *p* < .001, *η*^2^_G_ = 0.44] and group [*F*(1, 26) = 9.21, *p* = .005, *η*^2^_G_ = 0.22], consistent with our earlier analysis (Figure 2), but also for superordinate category [*F*(1, 26) = 234.17, *p* < .001, *η*^2^_G_ = 0.29]. Also consistent with before, there was a significant interaction between viewpoint and group [*F*(1, 26) = 15.33, *p* < .001, *η*^2^_G_ = 0.045], but there was no superordinate category interaction with either group or viewpoint. The three-way interaction was significant but with a small effect size [*F*(1, 26) = 4.82, *p* = .037, *η*^2^_G_ = 0.015]. Similar results were found with the untransformed data (main effects and interactions were significant or not) except for the three-way interaction. Indeed, Figure 4 shows no obvious three-way interaction: while some comparisons are clearly larger than others, the four columns are in the same rank order for the two superordinate categories. Thus, while there was an overall benefit for man-made over natural scenes there was little support for the notion that this varied across groups, viewpoints or their interaction.

Previous studies on man-made and natural gist categories have found (i) rapid and effective detection (Joubert et al., 2007) and discrimination (Furtak et al., 2022) of these superordinate categories, (ii) that superordinate image categorisation is superior to lower-level image categorisations (Loschky & Larson, 2010), (iii) that confusions across these two superordinate gist categories are fewer than within category (Loschky et al., 2015; see also below) and (iv) a modest processing advantage for man-made scenes over natural scenes (Joubert et al., 2007). Our results extend this picture by finding superior accuracy for the man-made category compared to the natural category. This suggests that diagnostic features might be more plentiful (for our set of images at least) in man-made scenes compared to natural scenes.

### Confusion Matrices for Scene Categorisation

**Figure 5.**
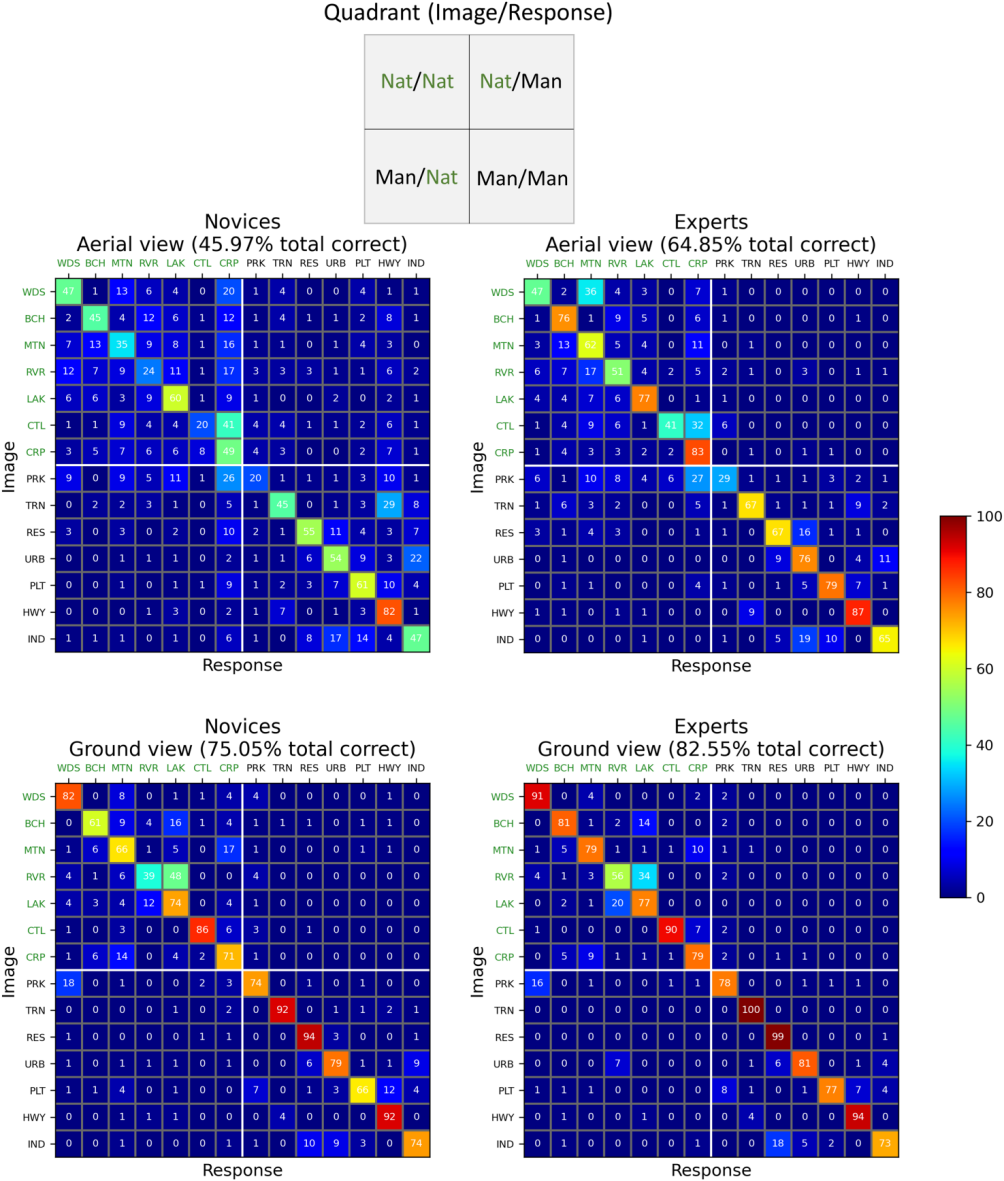
Confusion matrices (CMs) for the two groups (left, right columns) and the two viewpoint conditions (top, bottom rows). Cell entries are percentage responses per stimulus category (averaged across participants) rounded to integer values (i.e., each row sums to 100, deviations from this owing to rounding errors in this visualisation but not the calculations). These percentage scores are derived from 140 responses (14 participants × 10 exemplars) for each stimulus category in each panel. Within each CM, rows and columns indicate image and response categories, respectively. The natural- and man-made category divisions are denoted by labels in green and black, respectively (see online version of manuscript for full colour, including cell entries). The inset at the top of the figure summarises the layout of the four quadrants in each CM. (Nat = ‘natural’, Man = ‘man-made’). Axis label codes are: WDS: woods; BCH: beach; MTN: mountain/moor; RVR: river; LAK: lake; CTL: cattle field; CRP: crop field; PRK: park; TRN: train track; RES: residential buildings; URB: urban city; PLT: parking lot; HWY: highway; IND: industrial buildings.

To visualise our results in finer detail still, CMs were derived across the 14 × 14 image and response categories. These are shown in Figure 5 for the two groups (left and right columns) and two viewpoints (top and bottom rows). The categories are in the same rank order as in Figure 3. The inset at the top of Figure 5 summarises the 2 × 2 arrangement of the superordinate scene- and response-categories (natural and man-made) as conveyed by the four quadrants of each CM.

Here, we were interested in the pattern of confusions across categories, irrespective of the hit-rate per category. For example, if participants were biased towards making crop field responses, then we wanted the analysis to accommodate this; after all, the confusions are not performance measures of sensitivity. However, for completeness, in Appendix A we used a similar normalisation approach to our analysis above (in Figure 3d–f) to compensate for bias across the full CMs (cf., Loschky et al., 2015). The outcome of the inferential statistics in Appendix A was the same as what follows, indicating that normalisation or otherwise was not critical for our conclusions about confusions.

### Preliminary Observations

Within each CM in Figure 5, the main diagonal (top-left to bottom-right) indicates the percentage of correct responses (c.f. Figure 3a and b), and entries off this diagonal are confusion percentages. As to be expected, the CMs convey the earlier observations from Figure 2: novices made more confusions (i.e., mistakes) than experts (left panels vs. right panels), and confusions were more numerous for aerial-views than ground-views (top panels vs. bottom panels). However, most of the confusions tended to occur between sub-categories that were ranked as similar (along the natural-man-made dimension) in the preliminary survey (45% for Nat/Nat and 27% for Man/Man), placing them close to the main diagonal. The finding that most of the confusions were in the Nat/Nat category is consistent with the results of Loschky et al. (2015). Likewise, there were far fewer confusions crossing the natural and man-made categories (i.e., within the upper-right and lower-left quadrants of each plot) (10% for Nat/Man and 18% for Man/Nat). More generally, the confusions were spread quite thinly, and many did not occur (i.e., many cells have zero confusions in Figure 5) but also there was a handful of outliers: of the 728 possible confusions in Figure 5, only 28 of them (3.8%) had error-rates ≥12.5%, and only 8 of those (1.1%) had error-rates ≥25%. We return to this in a later section below.

### Mean Differences of Confusions Across CM Quadrants

Our main interest in Figure 5 was in potential differences for stimulus and response confusion categories both within and across participant groups and viewpoints (2 × 2). To investigate this at the superordinate level (the 2 × 2 inset of image-response categories in Figure 5), we compared number of confusions (by removing the cells on the main diagonals; c.f. Loschky et al., 2015) and performed 16 (2 × 2 × 2 × 2) non-parametric tests (Wilcoxon rank and Mann–Whitney *U*, as appropriate) for comparable quadrant-pairs across CMs (Table 1). (Shapiro–Wilk tests for normality pointed us away from performing parametric analyses.) We then applied Holm corrections to give conservative estimates of significance. The mean difference entries in Table 1 compare the number of confusions made by the average observer for each quadrant comparison-pair. (Note that the entries in Table 1 are for totals, whereas the entries in the CMs (Figure 5) are expressed in percent.)

**Table 1.** Statistical comparisons (Mann–Whitney *U*, Wilcoxon rank) of average observer confusions within quadrants across left/right and top/bottom panels in Figure 5 (excluding the main diagonals [correct responses] from the four panels).

Block	Comparison	Mann–Whitney *U* and Wilcoxon rank tests	Cell-by-cell
Group	Viewpoint	Quadrant	Group	Viewpoint	Quadrant	Mean diff.	SE	*U*/*W*	*p*	*p*(Holm)	RMS of diffs
1	Novices	Aerial	Nat/Nat	Experts	Aerial	Nat/Nat	7.93	2.77	45.0	.015*	N.S.	8.17
1	Novices	Aerial	Nat/Man	Experts	Aerial	Nat/Man	7.79	2.63	18.0	<.001***	0.004**	3.54
1	Novices	Aerial	Man/Nat	Experts	Aerial	Man/Nat	2.00	2.95	95.0	.908	N.S.	3.44
1	Novices	Aerial	Man/Man	Experts	Aerial	Man/Man	8.71	3.09	43.5	.013*	N.S.	6.53
2	Novices	Ground	Nat/Nat	Experts	Ground	Nat/Nat	6.86	2.52	46.5	.019*	N.S.	4.86
2	Novices	Ground	Nat/Man	Experts	Ground	Nat/Man	0.57	0.75	94.0	.869	N.S.	0.88 $
2	Novices	Ground	Man/Nat	Experts	Ground	Man/Nat	1.14	1.28	89.0	.693	N.S.	1.71
2	Novices	Ground	Man/Man	Experts	Ground	Man/Man	1.93	1.80	76.0	.321	N.S.	2.77
3	Novices	Ground	Nat/Nat	Novices	Aerial	Nat/Nat	−12.07	1.84	1.50	.001**	0.015*	13.69 £
3	Novices	Ground	Nat/Man	Novices	Aerial	Nat/Man	−7.86	2.27	0.00	.001**	0.014*	3.51
3	Novices	Ground	Man/Nat	Novices	Aerial	Man/Nat	−7.86	1.78	0.00	.001**	0.013*	6.60
3	Novices	Ground	Man/Man	Novices	Aerial	Man/Man	−12.93	1.97	0.00	.001**	0.012*	8.47
4	Experts	Ground	Nat/Nat	Experts	Aerial	Nat/Nat	−11.00	1.63	0.00	.002**	0.020*	12.94 £
4	Experts	Ground	Nat/Man	Experts	Aerial	Nat/Man	−0.64	0.57	20.00	.260	N.S.	1.20 $
4	Experts	Ground	Man/Nat	Experts	Aerial	Man/Nat	−7.00	1.09	0.00	.001**	0.015*	6.75
4	Experts	Ground	Man/Man	Experts	Aerial	Man/Man	−6.14	1.20	2.50	.003**	0.027*	6.58

Quadrants are labelled according to image category (Nat = ‘natural’, Man = ‘man-made’). Blocks 1 and 2 compare novices and experts for aerial and ground views, respectively, using the Mann–Whitney *U* test (*U*). Blocks 3 and 4 compare ground and aerial views for novices and experts, respectively, using the Wilcoxon rank test (W). Four pairs of quadrants are compared within each block. The inferential statistics for the differences were calculated for distributions of observer confusion totals across cells. SE: standard error. N.S.: Not significant. *p ≤ .05, **p ≤ .01, ***p ≤ .001. The final column is the root-mean-square (RMS) of the differences of cell values in the quadrant pairs for that row (excluding main diagonals as appropriate) which provides an index of inconsistency (lower values indicate greater consistency). The entries in this column were converted from percentages (in the CMs) to totals, making their units comparable to the mean difference column. $: the two smallest RMS quadrant differences (these are for the same quadrant across group and viewpoint). £: the two largest RMS quadrant differences (these are for the same quadrant across viewpoint for both groups).

Block 1 and 2 entries in Table 1 make comparisons across participant groups for aerial- and ground-views, respectively. Only the Nat/Man comparison for the aerial views (Block 1) was significantly different. These are the top-right quadrants for the two CMs on the top row of Figure 5. This means that when novices were more prone to confusion than experts (Figure 2), this tended to be for aerial views, where natural scenes were mis-categorised as man-made (e.g., categorising a beach as a highway), though see Appendix A for further analysis.

Block 3 and 4 entries in Table 1 make comparisons across viewpoints for novices and experts, respectively. These are quadrant comparisons across top and bottom rows in Figure 5. There were significant differences for all four quadrant comparisons for the novices (Block 3) and for three out of four for the experts (Block 4). The only exception was for Nat/Man for the experts. This means that for both groups, the increase in confusions when going from ground to aerial views was spread across the four logical possibilities, the exception being that experts did not increase their attribution of man-made categories to natural stimuli (top right quadrants in the right column panels).

### Consistency and Inconsistency of Confusions Across CM Quadrants: It's the Images That Matter

The formal analysis above compares mean participant differences of confusion scores across quadrant pairs but does not consider the consistency of those confusions. We addressed this here by calculating the RMS (equivalent to the standard deviation) of the paired cell differences (excluding the main diagonals from the four panels) for each of the 16 rows in Table 1 (e.g., in row 1, WDS/BCH for novices/aerial was paired with WDS/BCH for experts/aerial, and so on for the 42 cell pairs in those two quadrants). The results are shown in the right-hand column of Table 1 (in comparable units to the mean difference column). The greater values represent greater inconsistency across quadrant pairs, the largest two (£ in Table 1) being for Nat/Nat comparisons across viewpoint for each of the two groups (top left-hand quadrant pairs in Figure 5). This means that the greatest changes in confusion patterns were across viewpoint and within the natural category. Furthermore, this was similar across groups, indicating that expertise provided little protection for the influence of viewpoint in this sense. In contrast, the lower RMS values represent high consistency across quadrant pairs, the best two ($ in Table 1) being for Nat/Man comparisons at the ground level for the experts (i) across viewpoint and (ii) against novices (top right-hand quadrant pairs in right column and bottom row in Figure 5), presumably because there were few confusions in these quadrants overall.

**Figure 6.**
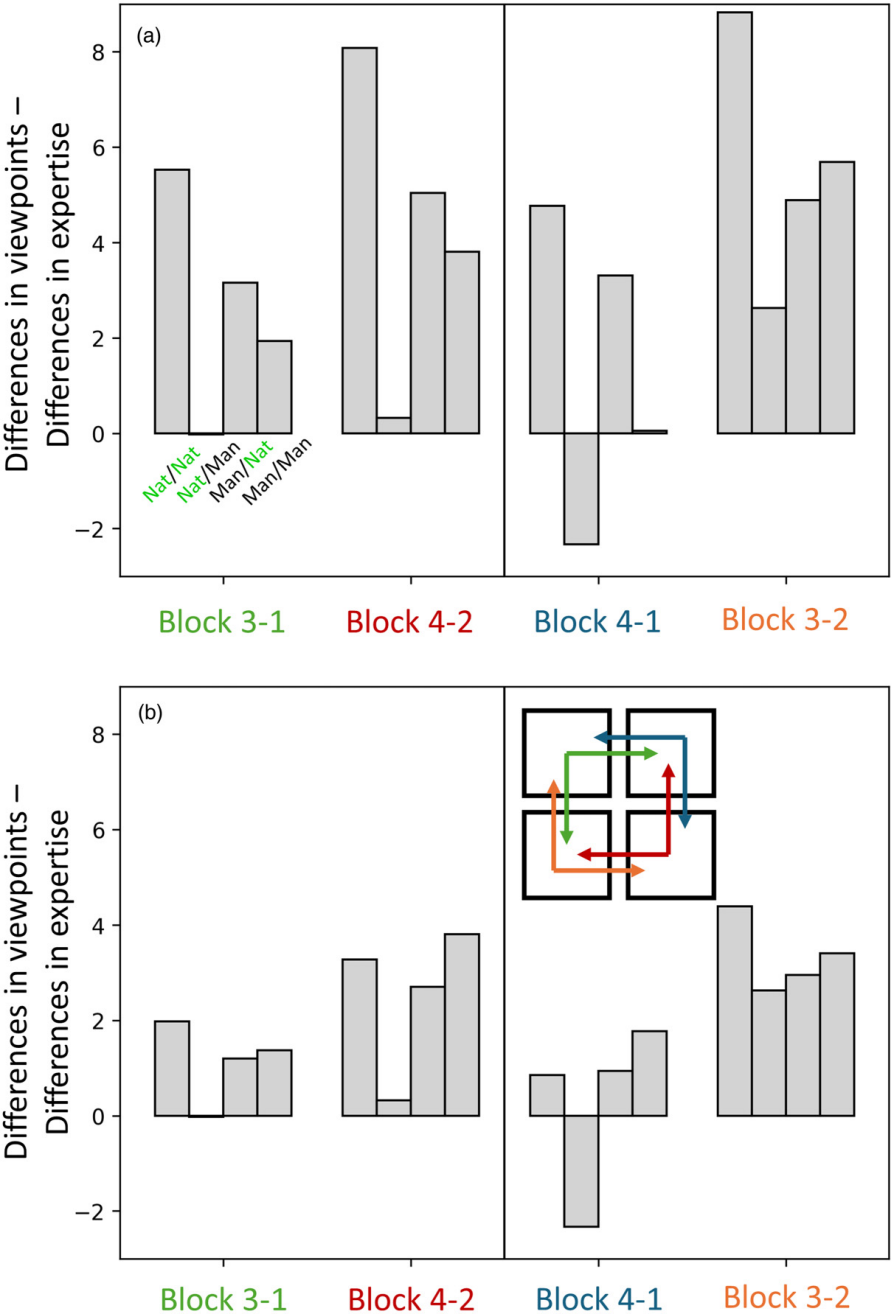
Quadrant comparisons for the RMS of confusions comparing changes in viewpoint with changes in expertise. Panel (a) is for the full set of confusions in Figure 5 (with the main diagonals [correct responses] removed) and the RMS differences listed in Table 1 (see also Table 2 for the steps to constructing this figure). The icon inset in the lower right of the figure illustrates the comparisons (of comparisons) with reference to the layout of the CMs in Figure 5. The colours in the icon relate to those in the *x*-axis labels and the ‘blocks’ are those in Table 1 (see online version of manuscript for full colour). Thus, Block 3-1 (green), takes an aerial viewpoint for novices (corner of green arrow in icon) and compares a change in viewpoint (vertical green arrow; Block 3) with a change in group (horizontal green arrow; Block 1). It does this for each of the four quadrants in the CM (see illustrative green and black labels beneath the first set of grey bars, upper left). Block 4-2 (red) deals with the complementary comparisons, starting with a ground view for experts. The righthand subpanel shows an alternative set (blue and orange) of paired arrangements of comparisons, completing the logical set. Positive values indicate that consistency (lower RMS differences) is greater across groups than across viewpoints and vice versa for negative values. Panel (b) is the same as Panel (a) but with the eight largest confusions (≥25% error rate) removed, illustrating that the general pattern of results is robust against outliers.

Next, we extended our descriptive observations of the (first-order) RMS differences to ask whether there was more consistency in confusions across participant groups or across viewpoints in general. To do this, we compared the RMS difference values (Table 1) for Blocks 1 and 2 against Blocks 3 and 4, as outlined in Table 2. There are two logical arrangements of these second-order comparisons to consider, as depicted by the icon of squares and arrows (red and green and blue and orange) in Figure 6 (the icon applies to both panels but is placed in panel b for convenience; see online version of manuscript for full colour). For example, starting with novices/aerial (top left panel in Figure 5), we can compare these four quadrants with experts/aerial (Block 1 in Table 1; a change in group) and also novices/ground (Block 3 in Table 1; a change in viewpoint), and then plot the differences of those two comparisons (green arrows in Figure 6b and the first set of four bars in Figure 6a; see also the top four rows of Table 2). Similarly, we can start with experts/ground (bottom right panel in Figure 5) and make four further quadrant comparisons for a change in group against a change in viewpoint (red arrows in Figure 6b and the second set of four bars in Figure 6a; see also the fifth to eighth rows of Table 2). However, the two starting points in these comparisons are arbitrary; we can start instead with experts/aerial and novices/ground (blue and orange arrows in Figure 6b) to generate eight further second-order comparisons (the righthand part of Figure 6a; see also the bottom half of Table 2).

**Table 2.** Derivation of the 16 differences of differences shown in Figure 6a derived from entries in Table 1.

Blocks in Table 1	Rows in Table 1	First RMS difference (from Table 1)	Second RMS difference (from Table 1)	Difference of RMS differences (Figure 6a)
**3-1**	9-1	13.69	8.17	5.52
**3-1**	10-2	3.51	3.54	-0.03
**3-1**	11-3	6.60	3.44	3.16
**3-1**	12-4	8.47	6.53	1.94
**4-2**	13-5	12.94	4.86	8.08
**4-2**	14-6	1.20	0.88	0.32
**4-2**	15-7	6.75	1.71	5.04
**4-2**	16-8	6.58	2.77	3.81
**4-1**	13-1	12.94	8.17	4.77
**4-1**	14-2	1.20	3.54	-2.34
**4-1**	15-3	6.75	3.44	3.31
**4-1**	16-4	6.58	6.53	0.05
**3-2**	9-5	13.69	4.86	8.83
**3-2**	10-6	3.51	0.88	2.63
**3-2**	11-7	6.60	1.71	4.89
**3-2**	12-8	8.47	2.77	5.7

The first two rows identify the block and row differences in Table 1 that produce the entries in the third and fourth columns here. These are the RMS differences in the last column of Table 1, each entry appearing twice within this table. The final column is the signed difference between the third and fourth column here and plotted in Figure 6a.

The direction of the differencing in the analysis above means that the signs of the bars in Figure 6a tell us whether consistency was greater for comparisons across participant groups or viewpoints. In most cases, the bars are positive meaning that consistency was greatest across participant groups. [The two exceptions (see the far-right column in Table 2) were for difference magnitudes in the lower tertile and prompted no obvious insights.] The overall pattern of the analysis did not change (Figure 6b vs. Figure 6a) when we removed the eight ‘large’ confusions discussed in the next section, indicating that our conclusion here does not rely on large outliers.

### Informal Considerations of Large Confusions

Further to the formal analyses above, there are several isolated confusions in Figure 5 worthy of note. We chose an arbitrary ≥25% error rate as a classification for large confusions, of which there were eight. The first is for the aerial view by novices (upper left plot, lower right quadrant) where train track images (TRNs) were often mis-categorised as highways (HWY) (29% errors compared to 9% for experts). Neither group made many confusions for this pairing at the ground level (2% and 0%). Presumably, this is because the distinguishing features are less readily discerned when viewed from afar (as in above) (see Figure 1), but the experts have learned to use the relevant cues for this important category in their professional work. This comparison is the main example for where experts were systematically less confused than novices.

Another source of confusion from the aerial view for novices (upper left plot, upper left quadrant) was the mis-categorisation of cattle fields (CTL) as crop fields (CRP) (41% errors). This confusion was also made by the experts (32%), though neither group made so many errors at the ground level (6% and 7%). Presumably, this is because the cattle were the only distinguishing feature across these categories and when viewed from afar (as in above), they were difficult to resolve, even for experts (see Figure 1). (Note that unlike the train track/highway distinction, experts do not routinely classify fields as crop or cattle fields but classify both as agricultural land.)

A situation we assume to be related to the one above is the mis-categorisation of the aerial views of parks (PRK) as crop fields (CRP), which was 26% for novices and 27% for experts (top row plots, lower-left quadrant) though far fewer errors were made at the ground level (3% and 1%). Again, the distinguishing man-made features of the park were probably too small to be reliably identified when viewed from afar (as in above) for either participant group (see Figure 1).

A fourth large confusion for aerial views was that experts mis-categorised woods (WDS) as mountain/moor (MTN) (36%). This error was worse than for the novices (13%) (top row plots, upper left quadrants) and poses something of a puzzle. We wondered whether this might relate to the dual terms (mountain and moorland) in the MTN label, but this seems unlikely given that OS experts are trained to discriminate between different vegetation and ground types including tree-cover (separating coniferous, non-coniferous and orchard), marsh, heath, rough grassland, rock and boulders and should therefore be sensitive to the cues that distinguished woods from both moorland and mountains. However, our categories did not directly map the fine sub-categories used by the OS and we speculate that experts—more than novices—may have associated trees with mountainous areas given the frequent use of lower slopes (vs. valley floors) for forestry in the UK.

A last result of note is the sole large confusion at the ground level, where rivers (RVR) were mis-categorised as lakes (LAK) for both novices (48%) and experts (34%) (bottom row plots, upper-left quadrants); these errors were much less for aerial views (11% and 4%) (top row plots, upper-left quadrants). Presumably, this is because the size and shape of the water mass are important features for this categorisation, and these are more readily discerned when viewed from afar and above (i.e., the features in these aerial scenes are particularly diagnostic for the task; Loschky et al., 2015). Indeed, for both groups we note that target sensitivity (% correct) was also better for lakes when the viewpoint was switched to above (Figure 3d and e), though this was significant only for the experts [*t*(13) = 3.627, *p* = .003]. This was the only category for which we have evidence for a benefit for an aerial viewpoint; rivers, for example, did not reap the same rewards. We note that this contrasts with Loschky et al. (2015) who found an aerial benefit for both rivers and lakes (both pre- and post-normalisation). The reason for this difference is not clear, but presumably lies in the details of the different images used across the two studies. We further note that Loschky et al. (2015) also found an aerial sensitivity benefit for one of their man-made categories, stadiums, for which we had no counterpart in our study.

Finally, and as mentioned above, we note that for studies where comparisons are made across ground- and aerial views (including ours) the aerial views are typically more distant. Thus, the change in diagnostic value of features for scenes when seen from above (be they diminished, as in cattle fields (CTL) or enhanced, as in rivers (RVR) might owe as much to a change in scale as a change in viewing angle.

## General Discussion

### Study Overview

In a rapid visual categorisation task with ground-view and aerial images of landscape scenes, expert surveyors trained on classification of aerial landscape images achieved greater accuracy than novices for aerial but not ground-view images. This extends previous work on expertise for aerial images (Bianchetti, 2016; Borders et al., 2020; Lloyd et al., 2002; Rhodes et al., 2021; Skog et al., 2024; Šikl et al., 2019), perceptual expertise in general (e.g., Bertram et al., 2013; Chin et al., 2018; DiGirolamo et al., 2023; Evans, Cohen, et al., 2011, 2013; Golan et al., 2014; Hussain, 2020; Ivy et al., 2023; Nodine & Krupinski, 1998; O’Neill et al., 2011; Vallée et al., 2024) categorisation of aerial images in general (Loschky et al., 2015), viewpoint dependency in general (e.g., Biederman & Gerhardstein, 1993; Lawson, 1999; Pannasch et al., 2014) and scene categorisation and gist perception in general (e.g., Evans, Horowitz, et al., 2011; Fu et al., 2016; Furtak et al., 2022; Greene & Oliva, 2009; Hansen & Loschky, 2013; Loschky & Larson, 2010; Mack & Clarke, 2012; Oliva & Torralba, 2006; Thorpe et al., 1996). More specifically, the benefit of expertise here is consistent with interventionist work in which novices have been trained on aerial images (Borders et al., 2020) and on medical images (DiGirolamo et al., 2023; Sha et al., 2020) thereby improving their performance.

With the caveat that our observations are necessarily attached to the specific images we used (280 in total, but only 10 in each viewpoint specific category), we have made the following main observations. Both experts and novices were better with ground-views than aerial-views, presumably because ground-view images are generally richer in diagnostic spatial structure (Loschky et al., 2015; Oliva & Torralba, 2001). Experts and novices made similar confusions in ground-view images but for aerial views, novices had a greater but weak tendency to making man-made responses to natural scenes. Overall, the greatest confusions were typically made from aerial views and by novices. However, when (presumably) key discriminatory features were small in the image (e.g., cattle), experts were also compromised for the distant aerial views. Contrariwise, when key discriminatory features were spatially extended (for rivers and lakes), both experts and novices made fewer specific category confusions (between rivers and lakes) from the more distant aerial views, though correct categorisation for these images benefitted from this elevated viewpoint only for experts and lakes. More generally, while overall performance deteriorated when viewpoint switched from the ground level to aerial, the pattern of hits and confusions across image categories also changed for both groups, presumably owing to changes in various diagnostic stimulus specific features. We found a modest benefit for the superordinate man-made category over natural scenes in the form of better normalised classification performance for scenes in the man-made category. In a man-made versus natural image classification task, Joubert et al. (2007) found that for their shortest response times, man-made responses were faster than natural responses. However, to our knowledge, our study is the first to find a classification accuracy benefit within the superordinate category for man-made scenes over natural scenes. Joubert et al. suggested that their result might owe to a greater number of diagnostic features in man-made scenes compared to natural scenes, and this might also account for our result here. However, there was little evidence that our man-made effect was modulated by viewpoint or group.

Perhaps the most striking observation is that despite the expected superiority of experts for the aerial viewpoint and a handful of idiosyncratic findings (discussed in the previous section) our main pattern of results—successful identifications, response biases, confusions, influences of subordinate category, influences of viewpoint—is remarkably similar across experts and novices: it is the image that matters (subordinate category and viewpoint), much less the expertise.

### Experimental Considerations

The observation above contrasts sharply with our previous work on the perception of 3D relief in noisy aerial images (Skog et al., 2024) where experts and novices adopted very different—and in some cases, tacit—strategies for discriminating hedges from ditches in a CI paradigm. Specifically, experts relied heavily (though not exclusively) on binocular disparity whereas novices (who were no less stereo-acute) tended to use luminance cues and an assumption of lighting from above. Notably, the OS experts in that study (and this), use (stereo) aerial photographs in their workplace which are taken in the northern hemisphere. There, sunlight is from the south, which means that a conventional image presentation of north at the top (as is OS practice) contravenes the visual system's typical assumption of lighting direction. This had a profound impact on the psychophysical behaviour of those experts, whose results showed a tacit assumption of lighting direction bias that was diminished or opposite to that of the novices. Whether lighting direction (which was from the south in the OS aerial images used here) was a factor in this study (which did not use binocular disparity) is unclear, though our casual observations during pilot testing suggested not (e.g., none of the aerial images flipped in depth for us with changes in orientation on the screen). Nonetheless, it is notable that the differences across novices and experts for the scene gist task here were modest by comparison to our work on 3D relief (Skog et al., 2024). In that work, the difference between experts and novices was largely one of kind, here it was largely one of degree.

The experts in our study were specifically proficient in making judgements on aerial images. However, (i) they were not familiar with the very brief backward masked presentations used here (100 ms each for contiguous presentations of target and mask stimuli) and (ii) the image category labels we chose were not those used in their workplace. Therefore, it is notable that their expertise from free viewing transferred to our psychophysical task. This is important because it validates our observation that the aerial expertise did not transfer to ground-based views (even though there was enough headroom to have done so in principle).

One aspect of scene categorisation studies that typically goes unremarked is the fidelity of the link between linguistic labels and the stimuli they describe. Labels such as smaller and bigger are unambiguous and easily described to naive participants, but labels such as river and lake or desert and field are more likely to conjure semantic confusions, for some participants at least. Such is the problem when using natural/man-made objects and scenes as visual stimuli. It is impossible to know from the data whether this issue taints work of this kind in general, including the work here, but this potential source of interference with the results should be kept in mind.

### The Nature of Expertise for Gist Perception

Our results provide strong support for the benefit of expertise in the perception of gist in aerial images, but what is it about perception that expertise changes? One view (Wolfe et al., 2011) is that our immediate impression of a scene involves parallel processes. One is a rapid statistically based global process with poor spatial resolution (e.g., Ventura et al., 2023) but a facility for sifting the parts to identify gist (what belongs) and outliers (what does not). It is a jumbled ‘bag of bits’. The other is more spatially directed, has fine resolution and provides detailed analysis to build detailed models of meaningful components in the scene. However, whether the preliminary analysis of the ‘bag of bits’ prompts a directed or undirected search for outliers or even exists independently of localisation is controversial (Brennan et al., 2018; Carrigan et al., 2018, 2019; Carrigan et al., 2018; Evans et al., 2013, 2016). Furthermore, regardless of how radiographers perform their task, their mechanism(s) for finding outliers in the ‘bag’ might refine differently and independently of the mechanism(s) for identifying the perceptual category (the gist) of the ‘bag's’ contents. Without a better understanding of the general mechanisms involved in identifying perceptual categories and their outlying components, it is difficult to place our work in a theoretical framework. However, a distinct possibility is that with respect to gist, our experts simply improved their earlier novice strategies for aerial scenes owing to their experience in the workplace.

Another aspect of visual expertise is whether it transfers from the domain of expertise to elsewhere. The general view is that it does not (Evans, Cohen, et al., 2011; Nodine & Krupinski, 1998; see also Ivy et al. (2023) for similar conclusions with caveats), and our finding here—that expertise in categorising aerial views did not transfer to ground views—is consistent with this view. However, the generality of this conclusion is challenged by Hussain (2020) who found that expert radiographers had greater sensitivity (*d*’) than novices for detecting band-limited textures in spatial noise (an unfamiliar task). Whether this difference reflects generic benefits such as higher-level attention, mechanistic changes in early vision, including resilience to distraction (Kontsevich & Tyler, 1999), or both, or other, remains unclear, though the finding (Hussain, 2020) that sensitivity improved similarly over time for both novices and experts shows that the beneficial transfer of expertise was not saturated. Further work is needed but it might be that slow cognitive abilities such as serial search (Nodine & Krupinski, 1998) and consolidation of memory (Evans, Cohen, et al., 2011) are immune to transfer, whereas benefits in early visual processing prove to be less domain specific. This sits comfortably with our other work (discussed above) which found effects of expertise in early vision (Skog et al., 2024) though the questions (under this working hypothesis) of which experiences and how they benefit/influence the mechanisms of early vision remain to be addressed.

## Summary and Conclusions

Aerial images are more difficult to process than ground-view images, but evidence from expert remote sensing surveyors showed that some of this processing difficulty can be overcome with experience. In a rapid scene categorisation task, experts and novices performed comparably with ground-view images, but experts were more accurate than novices with aerial images. Nonetheless, most striking of all was that in the study of gist perception here, the various patterns of behaviour across stimuli owed more to the images than the observers’ expertise. Whether comparable performance across viewpoints is possible with more extensive experience than the average of seven years here remains an open research question.

## Figures and Tables

**Figure A1.**
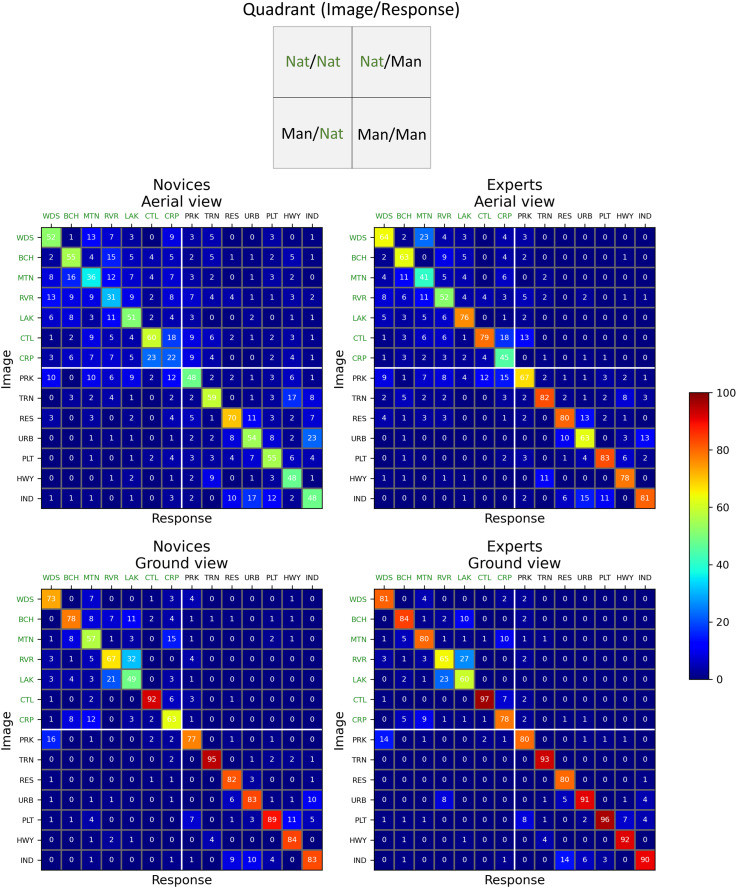
Normalised confusion matrices. The normalisation process here means that each column sums to 100, distinct from Figure 5 where each row sums to 100. See text and the caption of Figure 1 for details.

**Figure A2.**
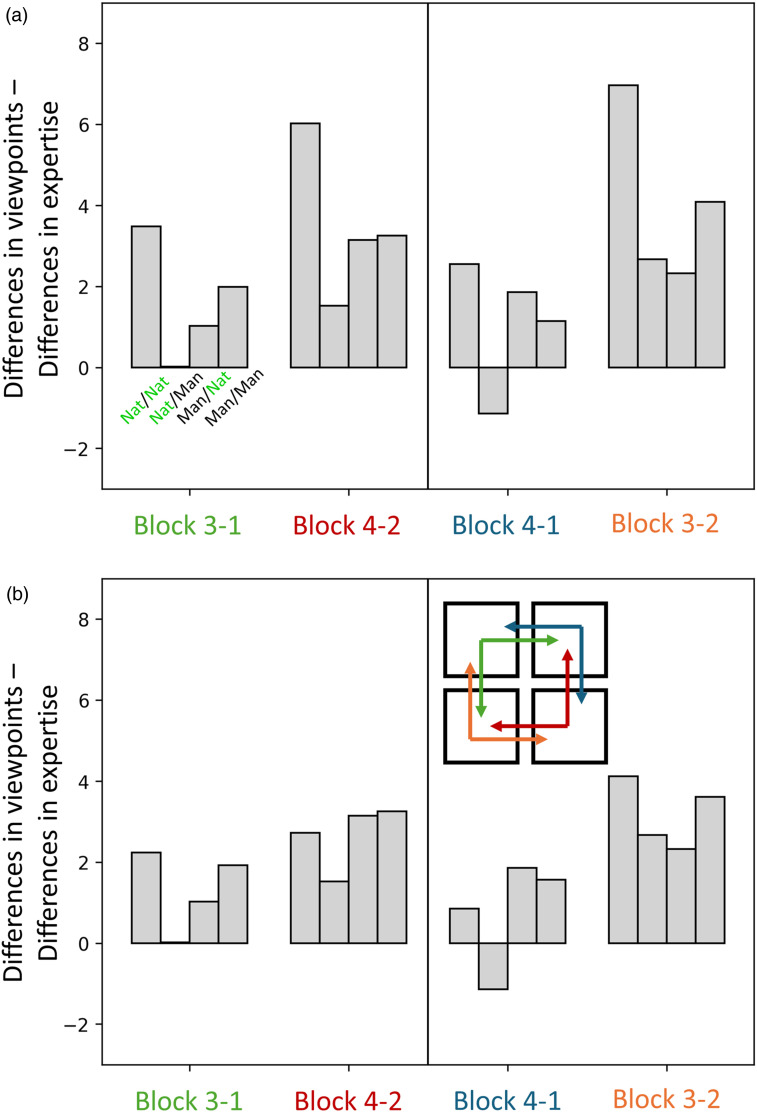
Quadrant comparisons for the RMS of confusions comparing changes in viewpoint with changes in expertise after normalisation of the confusion matrices. Panel (a) was derived from all of the confusion data in Figure A1 (excluding main diagonals). Panel (b) follows a similar procedure as in Figure 6 in that we removed the eight most outlying confusions from the analysis (this required a cut-off here of 18% compared to 25% in Figure 6). See the appendix text for details of the normalisation process and the caption of Figure 6 for further figure details.

## Notes

1. While potentially distinct in ground views, aerial views of the relatively gentle/low level mountains found in the UK were similar to moorland views, so these categories were merged.

2. Running PsychoPy online via Pavlovia is known to provide reliable visual timing durations, with variability and lag varying by only a few milliseconds across operating systems and web browsers (Bridges et al., 2020). We timed stimuli based on the system clock to provide approximately equal timing irrespective of varying monitor framerates. While this can be less accurate than counting frames, PsychoPy is careful to synchronise stimulus presentation with the monitor and will be accurate when the desired duration is an integer number of monitor frames. A 100 ms presentation time will occupy a whole number of frames for monitor framerates that are a multiple of 30 Hz. Twenty-six participants used monitors that met this requirement. One expert used a monitor with a framerate of 48 Hz and for one novice the framerate was 144 Hz, introducing timing errors of no more than 5%.

3. Response times were not analysed due to the requirement of mouse movements and clicks for responses.

## Appendix A: Confusion matrices and analysis for normalised results

The normalisation analysis here was performed following Loschky et al. (2015). Whether this approach or the analysis on the pre-normalised results (Figure 5 and Table 1) is more appropriate for our work depends on how the column biases are interpreted. If these are largely response biases, then the analysis here is more appropriate. If they are largely perceptual biases, then the earlier analysis is more appropriate. Our considerations in the main text pointed towards perceptual biases, though we could not rule out an interaction between response bias and viewpoint. Therefore, we performed the analysis here for completeness.

The CMs in Figure A1 are the same as those in Figure 5 except each cell has been normalised by dividing by the sum of the entries in each column and then multiplying by 100 to remove the column biases (e.g., compare the CRP column for aerial novices across Figure A1 and 5). This is the same normalisation process as for Figure 3d and e but applied to each cell. Table A1 provides the results for the same analyses as in Table 1 but applied to the normalised CMs of Figure A1. Some of the details in Table A1 differ from those in Table 1, but the overall picture is similar. First, the Holm corrected differences across quadrants are the same, except a swap in significance for Nat/Nat and Nat/Man in Block 1. This means that when novices were more prone to confusion than experts, this tended to be for aerial views, where confusions were made amongst natural scenes for the analysis here, rather than confusing natural scenes for man-made scenes by the earlier analysis.

**Table A1.** Statistical comparisons (Mann–Whitney *U*, Wilcoxon rank) of mean normalised confusions within quadrants across left/right and top/bottom panels in Figure A1 (excluding the main diagonals from the four panels).

Block	Comparison	Mann–Whitney *U* and Wilcoxon rank tests					Cell-by-cell
Group	Viewpoint	Quadrant	Group	Viewpoint	Quadrant	Mean diff.	SE	*U*/*W*	*p*	*p*(Holm)	RMS of diffs
1	Novices	Aerial	Nat/Nat	Experts	Aerial	Nat/Nat	10.93	1.73	27.5	0.001**	0.016*	6.69
1	Novices	Aerial	Nat/Man	Experts	Aerial	Nat/Man	6.29	1.20	43.5	0.013*	N.S.	3.59
1	Novices	Aerial	Man/Nat	Experts	Aerial	Man/Nat	0.14	0.78	89.5	0.710	N.S.	3.05
1	Novices	Aerial	Man/Man	Experts	Aerial	Man/Man	5.07	1.39	63.5	0.116	N.S.	4.59
2	Novices	Ground	Nat/Nat	Experts	Ground	Nat/Nat	5.21	1.11	45.5	0.016**	N.S.	3.21
2	Novices	Ground	Nat/Man	Experts	Ground	Nat/Man	0.64	0.38	94.5	0.887	N.S.	0.93 $
2	Novices	Ground	Man/Nat	Experts	Ground	Man/Nat	1.00	0.52	91.0	0.762	N.S.	1.75 $
2	Novices	Ground	Man/Man	Experts	Ground	Man/Man	1.86	0.76	77.0	0.344	N.S.	2.48
3	Novices	Ground	Nat/Nat	Novices	Aerial	Nat/Nat	−12.14	2.44	2.50	0.002**	0.026*	10.17 £
3	Novices	Ground	Nat/Man	Novices	Aerial	Nat/Man	−7.64	1.70	0.00	0.002**	0.026*	3.60
3	Novices	Ground	Man/Nat	Novices	Aerial	Man/Nat	−3.29	0.80	6.00	0.004**	0.040*	4.08
3	Novices	Ground	Man/Man	Novices	Aerial	Man/Man	−9.14	1.53	0.00	0.001**	0.016*	6.57
4	Experts	Ground	Nat/Nat	Experts	Aerial	Nat/Nat	−6.43	1.47	0.00	0.004**	0.040*	9.24 £
4	Experts	Ground	Nat/Man	Experts	Aerial	Nat/Man	−2.00	0.79	8.50	0.033*	N.S.	2.45
4	Experts	Ground	Man/Nat	Experts	Aerial	Man/Nat	−4.43	0.82	1.50	0.001**	0.015*	4.90
4	Experts	Ground	Man/Man	Experts	Aerial	Man/Man	−5.93	0.68	0.00	0.002**	0.024*	5.73

*Note*. Layout and symbols are the same as for Table 1.

A second change is that one of the two lowest RMS values ($, denoting high consistency between quadrants) has moved from the expert comparison for ground and aerial, Nat/Man (Block 4) to an expert comparison to novices at the ground level, Man/Nat (Block 2). As the RMS value (1.75) for this quadrant comparison is similar to the corresponding comparison in Table 1 (1.71), we do not attribute any significance to this other than to acknowledge that the normalisation process has lifted the floor and lowered the ceiling of consistency a little: the standard deviation of the RMS values in Tables 1 and A1 are, 3.9 and 2.5, respectively.

Following the procedure used in the main report for generating Figure 6, the RMS entries in Table A1 were used to produce Figure A2. The general patterns are very similar to those for the pre-normalised results in the main body of the report.
